# De novo 2q36.3q37.1 deletion encompassing *TRIP12* and *NPPC* yields distinct phenotypes

**DOI:** 10.1038/s41439-020-0107-1

**Published:** 2020-06-01

**Authors:** Yuto Kondo, Kohei Aoyama, Hisato Suzuki, Ayako Hattori, Ikumi Hori, Koichi Ito, Aya Yoshida, Mari Koroki, Kentaro Ueda, Kenjiro Kosaki, Shinji Saitoh

**Affiliations:** 1 Department of Pediatrics and Neonatology, Nagoya City University Graduate School of Medical Sciences, Nagoya, Japan; 2 Center for Medical Genetics, Keio University School of Medicine, Tokyo, Japan; 3 Department of Pediatrics, Japanese Red Cross Nagoya Daini Hospital, Nagoya, Japan

**Keywords:** Neurodevelopmental disorders, Growth disorders

## Abstract

We report a patient with developmental delay, extremely short stature, small hands, dysmorphic facial features, hearing loss, and epilepsy carrying a de novo 2.76-Mb deletion of 2q36.3q37.1, including *TRIP12* and *NPPC*. *TRIP12* haploinsufficiency causes developmental delay with isolated dysmorphic facial features, whereas *NPPC* haploinsufficiency causes short stature and small hands. This is the first report of a unique phenotype, which is secondary to a microdeletion encompassing *TRIP12* and *NPPC*.

Thyroid hormone receptor interacting protein 12 (TRIP12) is a human protein homologous to the E6-AP carboxyl terminus (HECT) domain E3 ubiquitin ligase and is involved in cell cycle progression and the maintenance of chromosome integrity^1^. *TRIP12* (MIM604506) haploinsufficiency has been reported to cause developmental delay, autism spectrum disorder (ASD), and facial dysmorphisms, which are collectively named Clark–Baraitser syndrome (MIM #617752)^2–4^.

C-type natriuretic peptide (CNP) plays an important role in cartilage homeostasis and endochondral bone formation and is encoded by the gene *natriuretic peptide precursor-C* (*NPPC*; MIM600296)^5^. *NPPC* variants have been reported to cause autosomal dominant idiopathic short stature with small hands^6^. Here, we describe a case of a 4-year-old boy with a de novo 2q36.3q37.1 deletion encompassing *TRIP12* and *NPPC* who presented with severe developmental delay, extremely short stature, small hands, dysmorphic facial features, hearing loss, and epilepsy.

The boy was born to healthy nonconsanguineous Japanese parents at 40 gestational weeks. His parents had no medical history. At birth, his weight, length, and occipitofrontal circumference (OFC) were 2710 g (−0.7 standard deviation (SD)), 46.6 cm (−1.1 SD), and 33 cm (−0.4 SD), respectively. Automated auditory brainstem response detection in a neonatal hearing screening test revealed a refer outcome. Urinal cytomegalovirus DNA was not detected in the neonatal period. Hearing threshold levels were bilateral sensorineural hearing loss of 70 dB at the age of 6 months and 30–40 dB at 4 years. He showed severe developmental delay and could hold up his head at 9 months, sit alone at 14 months, and stand with support at ~2 years. At 4 years, when this report was written, he was unable to speak or stand alone. He displayed behavioral abnormalities, such as aggression, repeatedly biting his intravenous drip line during hospitalization, and frequently striking his head against the wall. His short stature became apparent by 6 months of age (Fig. 1a). His height, weight, and OFC were 76.5 cm (−4.0 SD), 8.5 kg (−3.3 SD), and 50.5 cm (+0.9 SD) at 3 years, respectively, indicating relative macrocephaly (OFC more than +1 SD compared to height). We performed several examinations to evaluate his severe short stature. Complete blood count, blood chemistry, and thyroid function tests showed normal results. Insulin-like growth factor-1 was 46 mg/mL (−1.1 SD) at 3 years. His karyotype was 46, XY. A growth hormone stimulation test was not performed because his parents did not provide consent. He experienced four febrile seizures and two afebrile seizures at 2 years. His electroencephalogram showed frequent spike or spike-and-wave discharges in the left parietal region during drowsy and sleep states. He was diagnosed with epilepsy, and levetiracetam was started at 3 years. Head magnetic resonance imaging showed normal results. His facial features included a prominent forehead, mild hypertelorism, epicanthal folds, short nose, low hanging columella, short philtrum, everted vermilion of the upper lip, strabismus, and low-set ears (Fig. 1b, c), and his hands were small (Fig. 1d). After obtaining written informed consent, we performed array comparative genomic hybridization analysis and identified a de novo 2.76 Mb deletion (arr[GRCh37] 2q36.3q37.1(230377789_233136164)x1) encompassing 25 protein-coding genes; of these genes, *TRIP12* and *NPPC* are known to affect phenotypes attributable to autosomal dominant inheritance and cause developmental delay and short stature with small hands, respectively. Among the 25 genes, *TRIP12*, *NCL*, and *PSMD1* each have a “probability of being loss-of-function intolerant” (pLI) score of 1.0, indicating that these three genes are probably intolerant to a loss-of-function variation^7^. *NPPC* is classified as tolerant to loss-of-function variation because the phenotype caused by *NPPC* haploinsufficiency is not thought to confer significant survival or reproductive disadvantages. In addition, although abnormalities in *SP110*, *ARMC9*, *PDE6D*, and *DIS3L2*, which reside in the deleted region, have been reported to cause autosomal recessive inheritance diseases, our patient displayed different clinical features than those observed with these diseases^8^.

**Fig. 1:**
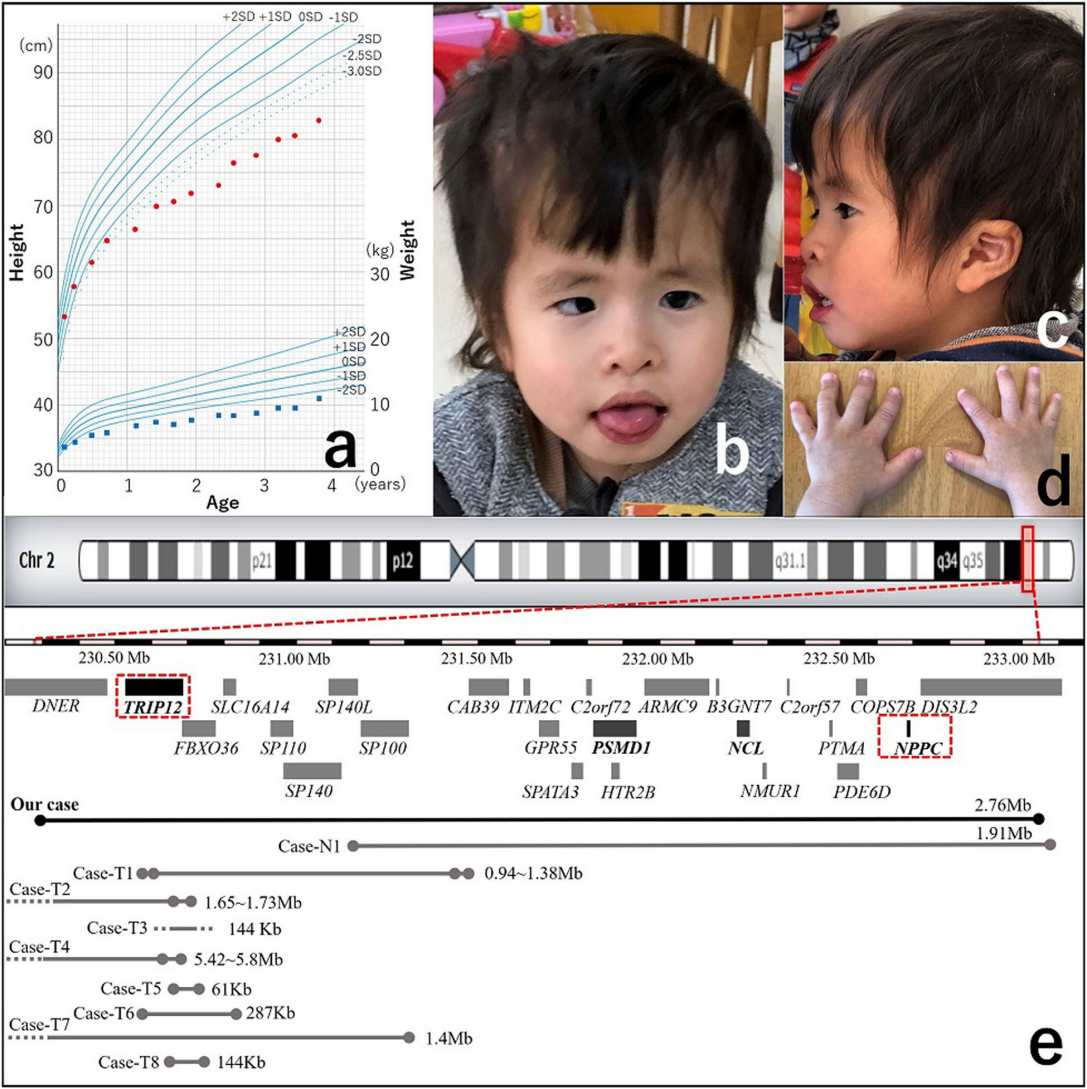
Growth curve, images of face and hands, and deleted genes in the patient. **a** The patient’s growth curve. **b**, **c** The patient exhibited prominent forehead, mild hypertelorism, epicanthal folds, short nose, low hanging columella, short philtrum, everted vermilion of the upper lip, strabismus, low-set ears, and relative macrocephaly at 4 years of age. We obtained written informed consent from the patient’s family for publication of this clinical report and accompanying images. **d** The patient presented with small hands. **e** Twenty-five genes in the 2.76-Mb 2q36.3q37.1 deletion (230377789_233136164) and deleted region of our case and previously reported copy number variant (CNV) deletion cases involving *TRIP12* or *NPPC* in Table 1.

Genetic findings related to *TRIP12* mainly include rare de novo variants, which have been identified from several large ASD or ID proband cohorts by whole-exome or target sequencing^9–14^. However, these reports did not describe detailed patient clinical information. Regarding clinical features, Bramswig et al. and Zang et al. reported 11 patients, including four previously reported cases^9,10,15^ and nine patients with inactivating single nucleotide variants or copy number variant (CNV) deletions in *TRIP12*, respectively, in 2017^2,3^.

CNP encoded by *NPPC* plays an important role in skeletal development. Additionally, *NPPC* alteration in humans was reported by Tassano et al. for the first time in a boy with short stature and several dysmorphic features; he had a 1.91 Mb 2q37 deletion, including *NPPC* (case-N1 in Fig. 1e and Table 1)^16^. Two *NPPC* variants were identified in six patients with short stature and small hands from only two families by Hisado-Oliva et al.^6^. They also showed that COP-7 cells transfected with *NPPC* variants detected in the families showed significantly reduced CNP-dependent cGMP synthesis even in the heterozygous state.

**Table 1:** Summary of CNV deletions including *TRIP12* or *NPPC* and adjacent genes.

Case	Deletion range chr2:	Deletion size	Gene^a^ number	Inheritance	Phenotype	Reference
Our case	230,377,789-233,136,164	2.76 Mb	25	de novo	Developmental delay, severe short stature, motor delay, epilepsy, bilateral hearing loss, relative macrocephaly, no words and not walk 49 months	This article
Case-N1	231,264,596-233,178,325	1.91 Mb	19	de novo	Development delay, cutaneous syndactyly of 2^nd^ and 3^rd^ toes bilaterally, peripheral hearing loss, relative macrocephaly	Tassano et al.^16^ Patient 1
Case-T1	230,489,478/230,513,445-231,457,431/231,508,839	0.98 ± 0.04 Mb	8	de novo	Developmental delay, normal height, microcephaly (25 percentile), obesity, unilateral hearing loss, Autism, first words 24 months, walk 20 months	Zhang et al.^3^ Subject 4
Case-T2	229,076,749/229,152,599-230,801,061/230,811,273	1.69 ± 0.04 Mb	4	de novo	Developmental delay, normal height, Autism, aggressive behaviors, 5–6 words 22 months, motor delay	Zhang et al.^3^ Subject 5
Case-T3	no data	144 Kb	2	Unknown	Developmental delay, motor delay, mild short stature (6 percentile)	Zhang et al.^3^ Suppl 2
Case-T4	225,043,475/225,360,778^b^-230,785,568/230,841,358^b^	5.61 ± 0.19 Mb	20	de novo	Intrauterine growth retardation, developmental delay, walk 22 months, profound speech deficit, normal height(+1 SD), large mouth, multiple renal cyst	Doco-Fenzy et al.^20^
Case-T5	230,778,385-230,839,009^b^	61 Kb	2	de novo	Autism, first words 12 months, first phrases 24 months, 5 years 9 months: global DQ 79	Pinto et al.^21^
Case-T6	230,689,285-230,976,668	287 Kb	3	de novo	Mild micrognathia, global developmental delay	DECIPHER ID 301556
Case-T7	230,020,617-231,444,802	1.4 Mb	9	de novo	Epicanthus, low columella, wide mouth, delayed speech and language development	DECIPHER ID 250590
Case-T8	230,724,038-230,868,128	144 Kb	2	Unknown	Cystic renal dysplasia, Global developmental delay	DECIPHER ID 281305

*DQ* developmental quotient.

^a^Protein coding genes.

^b^Genome coordinates differ from the original articles, which were converted from assembly NCBI36/hg18 to assembly GRCh37/hg19 by using Assembly Converter (http://grch37.ensembl.org/Homo_sapiens/Tools/AssemblyConverter).

*PSMD1* (proteasome 26S subunit, non-ATPase, 1, MIM617842) encodes a regulatory subunit of the 26S proteasome, which is a major ATP-dependent intracellular proteinase^17^. *NCL* (nucleolin, MIM164035) encodes a eukaryotic nucleolar phosphoprotein and is associated with the synthesis and maturation of ribosomes^18^. No specific phenotypes associated with *PSMD1* or *NCL* haploinsufficiency have been reported, whereas only one patient carrying a de novo *NCL* variant was identified by exome sequencing of 4293 families with developmental disorders^19^. Given that the deletion included four genes associated with autosomal recessive inheritance diseases, the major limitation of our study is that we did not analyze a hemizygous variation of the undeleted allele.

Eight CNV deletions smaller than 6 Mb involving *TRIP12* have been reported^3,20,21^. In addition, we identified five CNV deletions with phenotype information in the DECIPHER database v9.31^8^. None of these 13 CNVs included *NPPC*, *NCL*, or *PSMD1*, and five CNVs were intragenic deletions within *TRIP12*. The remaining eight deletions involved adjacent genes and *TRIP12* (case-T1–T8 in Fig. 1e and Table 1). For *NPPC*, case N1 was the only reported patient with a CNV deletion smaller than 6 Mb involving *NPPC*, and the deletion included *NCL* and *PSMD1* (case N1 in Fig. 1e and Table 1).

The clinical features associated with *TRIP*12 alterations have been previously summarized^2,3^. Clinical features such as developmental delay, hearing loss, and epilepsy in our patients could be attributed to *TRIP12* haploinsufficiency. Our patient showed epicanthic folds, hypertelorism, short nose, low hanging columella, and low-set ears, which partly overlapped with the dysmorphic facial features observed in patients with *TRIP12* alterations^2,3^. However, the low-set ears, prominent forehead, strabismus, short philtrum, everted vermilion of the upper lip, and relative macrocephaly in our patient were considerably similar to those in case N1. Case N1 had 19 deleted genes, including *NPPC*, *NCL*, and *PSMD1*, and all of them were included within the deletion range of our case. Recognizable facial dysmorphisms in patients with *NPPC* alterations have not been reported. Therefore, the characteristic facies in Case N1 and in our case is not explained by *NPPC* deletion but by other deleted genes, including *NCL* and *PSMD1*. Given the considerable facial similarity between Case 1 and our case, we speculated that facial dysmorphism in our case would be affected more by the 19 deleted genes than by *TRIP12* alteration.

Our patient showed developmental delay and short stature to a more severe extent than previously described for patients with *TRIP12* alterations. He was not able to speak any words or walk independently at 49 months of age, although previously described patients with *TRIP12* alterations such as case T1 and case T2 could speak their first words and walk independently at a mean age of 28 months (range 10–60, *n* = 15) and 20 months (range 13–29, *n* = 16), respectively^2,3^. Their heights were variable, and the mean height SD score was −0.3 SD (range −2.2 to +1.1, *n* = 16). Short stature and small hands in our patient were thought to be mainly affected by the *NPPC* deletion. However, *NPPC* alterations alone do not explain his extremely short stature. The mean height SD score of the six patients with *NPPC* heterozygous variants (−2.9 SD, range −4.3 to −2.3) was 0.9 SD lower than that of the five patients without *NPPC* variants (−2.0 SD, range −4.8 to −0.4) in the two reported families^6^. Importantly, case T4, with the largest deletion expanding in the centromeric direction, showed a normal height and moderate developmental delay (initial walk at 22 months). Thus, genes other than *TRIP12* and *NPPC* located in the deletion region might have contributed to the exaggerated phenotypes in terms of developmental delay and short stature. Because of extreme intolerance to loss-of-function variations, *NCL* and *PSMD1* could be good candidate genes for the exaggerated phenotypes.

In conclusion, we report a patient with a de novo 2.76-Mb deletion of 2q36.3q37.1 encompassing *TRIP12* and *NPPC*. This is the first report of a patient with a unique phenotype of combined *TRIP12* and *NPPC* haploinsufficiency. The severe developmental delay and extremely short stature of the patient imply that *NCL* and *PSMD1* are potentially disease-modulating genes.

## Data Availability

The relevant data from this Data Report are hosted at the Human Genome Variation Database at 10.6084/m9.figshare.hgv.2864; 10.6084/m9.figshare.hgv.2867.
